# Association of cholesterol, high-density lipoprotein, and glucose (CHG) index with chronic kidney disease in Chinese community adults: findings from the REACTION study

**DOI:** 10.3389/fnut.2026.1778139

**Published:** 2026-05-11

**Authors:** Xue Bai, Zujiao Wu, Qiuqing Wang, Ying Miao, Zhihong Zhang, Lilan Liu, Wei Huang, Fangyuan Teng, Yong Xu, Qin Wan, Pijun Yan

**Affiliations:** 1 Department of Endocrinology and Metabolism, The Affiliated Hospital of Southwest Medical University, Luzhou, China; 2 Metabolic Vascular Disease Key Laboratory of Sichuan Province, Luzhou, China; 3 Sichuan Clinical Research Center for Diabetes and Metabolism, Luzhou, China; 4 Sichuan Clinical Research Center for Nephropathy, Luzhou, China; 5 Cardiovascular and Metabolic Diseases Key Laboratory of Luzhou, Luzhou, China; 6 Department of Clinical Nutrition, Chengdu Eighth People’s Hospital (Geriatric Hospital of Chengdu Medical College), Chengdu, China; 7 Department of General Medicine, The Affiliated Hospital of Southwest Medical University, Luzhou, Sichuan, China; 8 Department of Endocrinology, Hejiang People’s Hospital, Luzhou, China

**Keywords:** biomarker, Chinese population, cholesterol, high-density lipoprotein, and glucose index, chronic kidney disease, community adults

## Abstract

**Background:**

While a limited-scale study in Turkey identified an association between the cholesterol, high-density lipoprotein, and glucose (CHG) index and diabetic nephropathy, evidence on the relationship between the CHG index and chronic kidney disease (CKD) is lacking in Chinese general population. This study aimed to investigate the association of CHG index with CKD in this specific demographic.

**Methods:**

A total of 9,095 Chinese participants aged ≥40 years were recruited from five regional communities of Luzhou city between May 2011 and December 2011. CHG index was calculated, and its possible relationships with CKD were evaluated by multivariate logistic regression analyses. Receiver operating characteristic (ROC) analysis was conducted to identify the predictive performance, and subgroup analysis evaluated its applicability in different populations.

**Results:**

The subjects with higher CHG index quartiles had significantly higher prevalence of CKD compared to those with lower quartiles (*P* for trend < 0.01). Multivariate logistic regression analysis demonstrated that per Standard deviation (SD) increase in CHG index remains significantly associated with a 57.7% increased risk of CKD [odds ratios (OR) = 1.577; 95% confidence intervals (CI) 1.301–1.911; *P* < 0.01], and subjects in the highest quartile of CHG index were significantly associated with a 32.8% increased risk of CKD when compared to those in the lowest quartile (OR = 1.328; 95% CI = 1.069–1.648; *P* < 0.01). Stratified analysis revealed that the associations between CHG index quartiles and CKD risk were observed only in subjects who were men, non-smoker, non-drinker, aged ≥60 years, receiving a less than high school education, having overweight/obesity, type 2 diabetes mellitus, dyslipidemia, normal blood pressure, and no atherosclerotic cardiovascular disease (*P* for trend < 0.01 or *P* for trend < 0.05). The optimal cutoff point for CHG index to distinguish patients with CKD from those without was 0.601, with a sensitivity of 73.6% and a specificity of 40.6%.

**Conclusion:**

The CHG index was closely associated with CKD, and might be a potential biomarker for CKD in Chinese community adults.

## 1 Introduction

Chronic kidney disease (CKD), characterized by abnormalities of kidney structure or function present for more than 3 months, represents a critical global public health issue. It is estimated that CKD affects approximately 10% of the world’s population (1), with prevalence rising steadily due to aging populations and increasing rates of diabetes and hypertension. The harms of CKD are profound, leading to devastating complications, including cognitive impairment, end-stage renal disease (ESRD) requiring dialysis or transplantation, accelerated atherosclerotic cardiovascular disease (ASCVD), and heightened mortality (1). Current diagnostic strategies, relying primarily on serum creatinine (Cr) and estimated glomerular filtration rate (eGFR), often detect the disease only after substantial and irreversible damage has occurred. Furthermore, while treatments such as RAAS inhibitors can slow progression, they cannot reverse established injury, and therapeutic options, particularly in advanced stages, remain extremely limited. Consequently, there is a pressing need to identify novel, reliable, and sensitive biomarkers that can detect CKD at its earliest, most treatable stages, enabling timely intervention to halt or prevent disease development and progression.

Compelling data indicate that insulin resistance (IR) and cardiometabolic disturbance play important roles in the development and progression of CKD (1, 2). The cholesterol, high-density lipoprotein, and glucose (CHG) index, a newer composite measure that integrates blood lipid and glucose levels, has been proposed as a marker of insulin resistance (IR) and cardiometabolic disturbance (3), and an elevated CHG index is significantly correlated with an increased risk of type 2 diabetes mellitus (T2DM), cardiovascular disease (CVD), stroke, and hypertension (3–8). Given the close associations between CHG index, IR, cardiometabolic disturbance, and CKD, it is plausible that the CHG index might be significantly associated with CKD. Recently, a cross-sectional study consisting of only 175 patients with a prior diagnosis of T2DM and a diabetes duration of more than 10 years showed that the CHG index was elevated in T2DM patients with diabetic nephropathy and was the only independent predictor of diabetic nephropathy (9). Moreover, IgA nephropathy (IgAN) patients with elevated CHG index exhibited more severe clinical (decreased eGFR, and elevated serum Cr and 24-h urine protein levels) and pathological profiles (tubular atrophy/interstitial fibrosis and renal arterial intimal thickening with hyaline degeneration), along with a shorter mean renal survival time (10). However, the relationship of CHG index with CKD has not yet been investigated in Chinese general middle-aged and elderly population.

For this reason, we evaluated the relationship between the CHG index and CKD in such population using data from the Risk Evaluation of cAncers in Chinese diabetic Individuals (REACTION) study.

## 2 Patients and methods

### 2.1 Study population

This study was drawn from the REACTION study, a nationwide, multicenter, population-based cohort study of 259,657 individuals aged 40 years or older from 25 communities across mainland China. The REACTION study was designed to investigate the association between cardiometabolic risk factors and specific clinical outcomes in the Chinese population. Detailed information on this study has been described previously (11, 12). Data for the present study were derived from five communities in Luzhou city, Sichuan Province. From May to December 2011, a total of 10,150 participants aged ≥40 years were recruited (13). Participants diagnosed with type 1 diabetes mellitus, those using glucose-lowering and lipid-lowering medications, and those with unreasonable or missing key data were excluded. Ultimately, 9,095 eligible participants were included in the final analysis.

The present study was approved by the Ethical Review Committee of Ruijin Hospital affiliated with Jiao-Tong University School of Medicine and by the Affiliated Hospital of Southwest Medical University. All methods were performed in accordance with the relevant guidelines. Written informed consent was obtained from all participants.

### 2.2 Data collection and measurements

Information on sociodemographic characteristics (sex, age, and education level), lifestyle behaviors (smoking status and alcohol consumption), and medical history was collected face-to-face by trained interviewers using a standard questionnaire. Education attainment was categorized as less than high school (<9 years) and high school or further (≥9 years). Participants were divided into three groups according to smoking status: never smokers, occasional smokers (less than one cigarette per day or less than seven cigarettes per week), and frequent smokers (at least one cigarette per day or almost every day) (14). Alcohol consumption was defined as follows: never, occasional (less than once a week), and frequent (at least once perweek during the previous 6 months) (15).

Body weight and height measurements were measured according to a standard protocol. Body-mass index (BMI) was calculated as weight in kilograms divided by height in meters squared. Systolic blood pressure (SBP), diastolic blood pressure (DBP), and heart rate (HR) were measured three times in a sitting position using an OMRON electronic blood pressure monitor, with an interval of 1 min between measurements after at least 5 min of rest. The average of the three readings was used for the final analysis.

All participants underwent a 75-g oral glucose tolerance test (OGTT) after an overnight fast of at least 10 h, with blood samples collected at 0 and 2 h. Fasting blood glucose (FBG), 2-h postload blood glucose (PBG), glycated hemoglobin A1C (HbA1c), total cholesterol (TC), triglyceride (TG), high-density lipoprotein cholesterol (HDL-C), low-density lipoprotein cholesterol (LDL-C), alanine aminotransferase (ALT), aspartate aminotransferase (AST), glutamyl transpeptidase (GGT), and Cr were measured according to relevant protocols and guidelines. All laboratory analyses were performed at a certified central laboratory located at the Affiliated Hospital of Southwest Medical University, which is accredited in accordance with ISO 15189.

Basal metabolic rate (BMR) was estimated using the Harris-Benedict equation: For men, BMR = 88.362 + (13.397 × weight in kg) + (4.799 × height in cm)−(5.677 × age in years); for women, BMR = 447.593 + (9.247 × weight in kg) + (3.098 × height in cm)−(4.330 × age in years) (16). The CHG index was calculated as Ln [TC (mg/dL) × FBG (mg/dL)/2 × HDL-C (mg/dL)] (3). The GGT to HDL-C (GGT/HDL-C), AST to ALT (AST/ALT), and HbA1c to HDL-C (HbA1c/HDL-C) ratios were also calculated.

### 2.3 Definition of CKD

The eGFR was calculated using the Japanese coefficient-modified Chronic Kidney Disease Epidemiology Collaboration (CKD-EPI) equation (8). In accordance with the National Kidney Foundation guidelines, CKD was solely defined as an eGFR < 60 ml/min/1.73 m^2^ (17). Most epidemiological studies on CKD are based solely on eGFR because albuminuria was tested much less frequently than creatinine. In the present study, we also did not consider combination of albuminuria and decreased eGFR as the definition of CKD urinary albumin-to-creatinine ratio (UACR) was not available for 1,981 participants.

### 2.4 Definition of other variables

Obesity was defined as a BMI of ≥28.0 kg/m^2^, according to the criteria for the Chinese population (13). Dyslipidemia was defined as TC ≥ 6.22 mmol/L, TG ≥ 2.26 mmol/L, HDL-C < 1.04 mmol/L, LDL-C ≥ 4.14 mmol/L, according to the National Cholesterol Education Program (NCEP) Expert Panel on Detection, Evaluation, and Treatment of High Blood Cholesterol in Adults (Adult Treatment Panel III; ATP III) (18, 19). According to the 2010 American Diabetes Association criteria, T2DM was defined as FBG ≥ 7.0 mmol/L, PBG ≥ 11.1 mmol/L, HbA1c ≥ 6.5%, or a self- reported previous diagnosis of diabetes by a health care professional. Among participants without diabetes, normal glucose tolerance (NGT) was defined as FBG < 5.6 mmol/L, PBG < 7.8 mmol/L, and HbA1c < 5.7%; prediabetes was defined as FBG 5.6–6.9 mmol/L, or PBG 7.8–11.0 mmol/L, or HbA1c 5.7%–6.4% (20). Hypertension was defined as SBP ≥ 140 mmHg, DBP ≥ 90 mmHg, or self- reported previous diagnosis of hypertension by clinicians and current use of antihypertensive medication (19). Among participants without hypertension, prehypertension was defined as SBP 120–139 mmHg or DBP 80–89 mmHg (21). Coronary heart disease (CHD) was defined as self-reported history of CHD, including myocardial infarction, angina, or coronary revascularization (22). Stroke was defined as a self-reported history of hemorrhagic and ischemic stroke (23). Peripheral arterial disease (PAD) was defined as a self-reported history of claudication, gangrene or ulceration, peripheral artery revascularization, or major amputation secondary to PAD (24). In accordance to the 2018 AHA/ACC cholesterol management guidelines, ASCVD was defined as a history of CHD, stroke, and symptomatic PAD (25).

### 2.5 Statistical analysis

Statistical analysis was performed using SPSS version 20.0 (SPSS Inc., Chicago, IL, USA). Kolmogorov-Smirnov test for normality and Levene’s homogeneity of variance test was conducted. Continuous variables are presented as mean ± standard deviation (SD), and categorical variables are presented as numbers (%).

For comparison of demographic, clinical and laboratory parameters among different groups, one-way analysis of variance (ANOVA) was used for normally distributed data, and the Kruskal-Wallis test was used otherwise. Categorical data were analyzed using the chi-squared test. The univariate logistic regression analyses were performed to determine the association of CHG index and other variables with the risk of prevalent CKD in Chinese general middle-aged and elderly population. Participants were then divided into quartiles based on the CHG index: Q1, CHG index ≤ 12.60; Q2, 12.60 < CHG index < 12.95; Q3, 12.95 ≤ CHG index < 13.25; Q4, CHG index ≥ 13.25. Logistic regression analyses with unadjusted and multivariate-adjusted models was performed to determine the association between CHG index quartiles and the risk of prevalent CKD. Model 1 was unadjusted; Model 2 was adjusted for gender and age; Model 3 was additionally adjusted for smoking, drinking, education level, BMR, and HR based on Model 2; Model 4 was additionally adjusted for ALT, AST, GGT, GGT/HDL-C, AST/ALT, overweight/obesity, hypertension, dyslipidemia, HbA1c/HDL-C, and T2DM based on Model 3. Model 5 was additionally adjusted for ASCVD based on Model 4. Odds ratios (OR) and corresponding 95% confidence intervals (CI) were calculated. Stratified analyses were conducted by sex (women vs. men), age (<60 vs. ≥60 years), smoking (no vs. yes), drinking (no vs. yes), education level (<9 vs. ≥ 9 years), BMI status (normal weight vs. overweight/obesity), glucose metabolism status (NGT, prediabetes, vs. T2DM), blood pressure status (normal blood pressure, prehypertension, vs. hypertension), dyslipidemia (no vs. yes), and ASCVD (no vs. yes). Stratified analyses were performed to explore the factors potentially influencing the relationship between the CHG index and CKD. Potential interactions between the CHG index and each stratifying variable were assessed using logistic regression analyses, with adjustment for sex, age, smoking, drinking, education level, BMR, HR, ALT, AST, GGT, GGT/HDL-C, AST/ALT, overweight/obesity, hypertension, dyslipidemia, HbA1c/HDL-C, T2DM, and ASCVD. Finally, receiver operating characteristic (ROC) curve analysis was performed to identify the optimal cutoff value of the CHG index for identifying CKD.

All *P*-value are two-tailed, and values < 0.05 were considered to statistically significant.

## 3 Results

### 3.1 Clinical and laboratory characteristics of the study population

**FIGURE 1:**
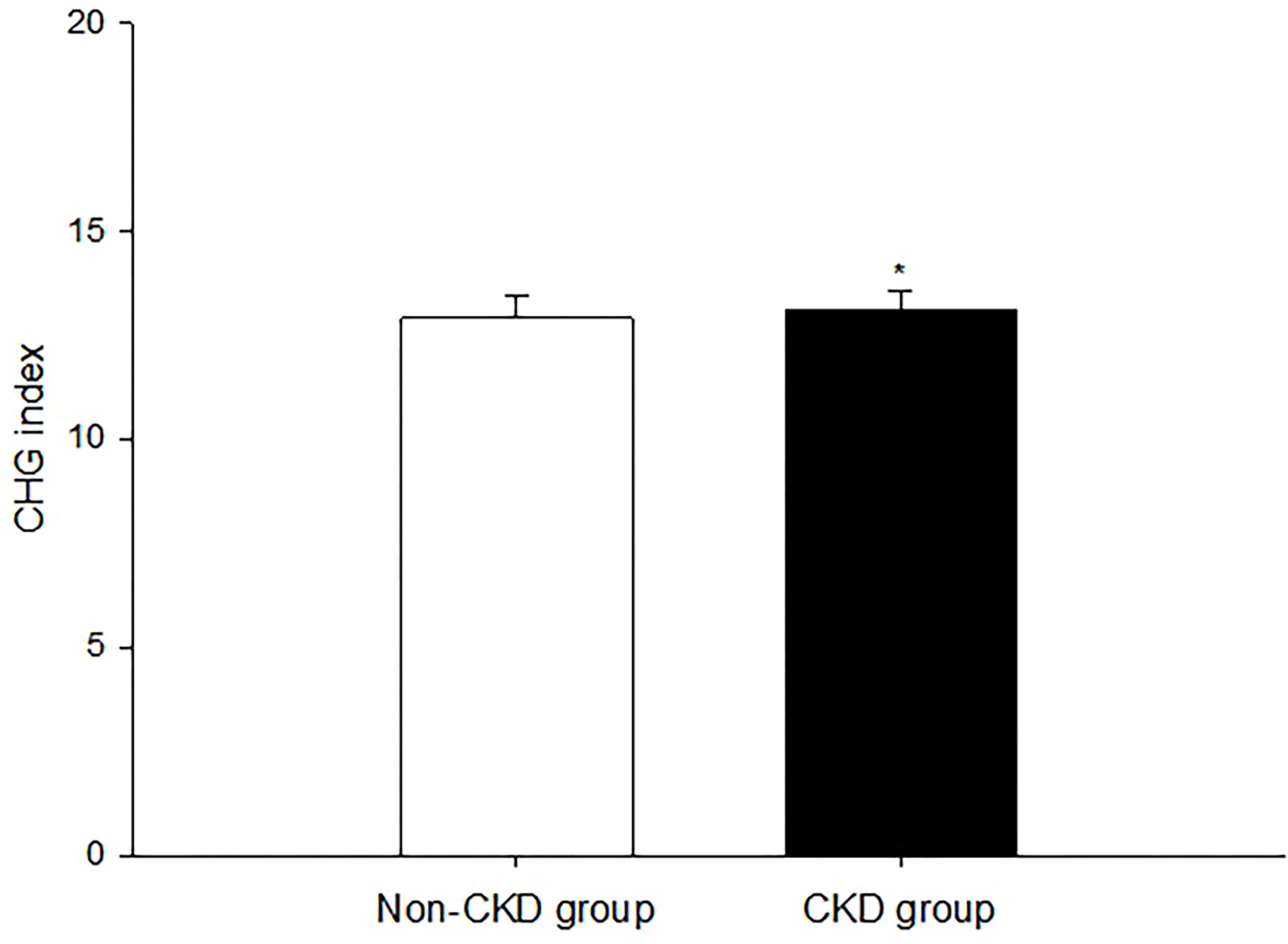
Level of cholesterol, high-density lipoprotein, and glucose (CHG) index between participants with chronic kidney disease (CKD) and without CKD in Chinese community adults. Vs. Non-CKD, *P < 0.01.

The basic characteristics of the study population according to CHG index quartiles are summarized in Table 1 and Supplementary Table 1. Among the 9,095 participants included in this study, 239 (2.63%) had CKD. The prevalence of CKD increased across CHG index quartiles: 1.67%, 2.23%, 2.79% and 3.81%, respectively. No significant differences were observed across CHG index quartiles in the proportion of frequent drinkers, DBP, the prevalence of hypertension, CHD, stroke, PAD, and ASCVD (all *P* > 0.05). Participants in higher CHG index quartiles were older, more likely to be never smokers and never drinkers, receive less than a high school education, and had higher HR, SBP, TC, TG, HDL-C, LDL-C, FBG, PBG, HbA1c, GGT/HDL-C, GGT, AST, ALT, AST/ALT, Scr, and higher prevalence of CKD and T2DM (all *P* for trend < 0.01). Conversely, participants in higher CHG index quartiles were less likely to be male, occasional and frequent smokers, occasional drinkers, and had lower BMR, HbA1c/HDL-C, eGFR, and lower prevalence of overweight/obesity, dyslipidemia, and prediabetes (*P* for trend < 0.01 or *P* for trend < 0.05). Participants with CKD had significantly higher CHG index compared to those without CKD (13.09 ± 0.49 vs. 12.90 ± 0.52, *P* < 0.01, Figure 1).

**TABLE 1:** Clinical and biochemical characteristics of study participants according to quartiles of the CHG index.

Variable	Total	Q1	Q2	Q3	Q4	*P-*value
	(*n* = 9,095)	(*n* = 2,275)	(*n* = 2,244)	(*n* = 2,291)	(*n* = 2,285)	
		≤12.60	12.61–12.94	12.95–13.24	≥13.25	
Male (*n*, %)	3,003 (33.02%)	922 (40.53%)	827 (36.85%)	735 (32.08%)	519 (22.71%)	<0.001
Age (years)	58.20 ± 10.20	57.66 ± 10.79	57.75 ± 10.33	58.08 ± 10.01	59.29 ± 9.55	<0.001
BMI (kg/m^2^)	23.97 ± 3.95	24.16 ± 4.23	24.07 ± 3.87	23.91 ± 3.75	23.74 ± 3.93	0.002
Smoking (*n*, %)						
Never	7,197 (79.13%)	1,707 (75.03%)	1,709 (76.16%)	1,829 (79.83%)	1,952 (85.43%)	<0.001
Occasional	372 (4.09%)	103 (4.53%)	111 (4.95%)	92 (4.02%)	66 (2.89%)	0.003
Frequently	1,526 (16.78%)	465 (20.44%)	424 (18.89%)	370 (16.15%)	267 (11.68%)	<0.001
Drinking (*n*, %)						
Never	6,031 (66.31%)	1,454 (63.91%)	1,485 (66.18%)	1,507 (65.78%)	1,585 (69.37%)	0.001
Occasional	2,104 (23.13%)	571 (25.10%)	503 (22.42%)	537 (23.44%)	493 (21.58%)	0.032
Frequently	960 (10.56%)	250 (10.99%)	256 (11.41%)	247 (10.78%)	207 (9.06%)	0.052
Education level (*n*, %)						
Less than high school	6,477 (71.21%)	1,553 (68.26%)	1,612 (71.84%)	1,635 (71.37%)	1,677 (73.39%)	0.002
High school or further	2,618 (28.79%)	722 (31.74%)	632 (28.16%)	656 (28.63%)	608 (26.61%)	0.002
SBP (mmHg)	124.48 ± 19.57	123.56 ± 18.82	123.87 ± 19.57	124.80 ± 19.59	125.68 ± 20.22	0.005
DBP (mmHg)	76.59 ± 10.83	76.80 ± 10.57	76.60 ± 11.01	76.63 ± 10.76	76.33 ± 10.99	0.457
TC (mmol/L)	4.59 ± 1.14	3.33 ± 0.69	4.31 ± 0.57	4.95 ± 0.63	5.77 ± 0.89	<0.001
TG (mmol/L)	1.57 ± 1.20	1.39 ± 1.20	1.62 ± 1.22	1.63 ± 1.20	1.64 ± 1.16	<0.001
HDL-C (mmol/L)	1.25 ± 0.35	0.89 ± 0.18	1.16 ± 0.17	1.35 ± 0.21	1.62 ± 0.31	<0.001
LDL-C (mmol/L)	2.59 ± 0.82	1.81 ± 0.48	2.41 ± 0.51	2.81 ± 0.61	3.30 ± 0.79	<0.001
FBG (mmol/L)	5.67 ± 1.27	5.37 ± 0.68	5.49 ± 0.81	5.59 ± 0.84	6.23 ± 2.04	<0.001
PBG (mmol/L)	8.47 ± 3.58	7.94 ± 2.64	8.10 ± 2.79	8.26 ± 3.07	9.56 ± 5.03	<0.001
HbA1c (%)	6.00 ± 0.88	5.88 ± 0.54	5.90 ± 0.63	5.94 ± 0.66	6.29 ± 1.35	<0.001
Cr(μmol/L)	64.67 ± 21.52	58.75 ± 23.58	65.08 ± 21.27	66.99 ± 22.68	67.86 ± 16.71	<0.001
eGFR (mL/min/1.73 m2)	95.48 ± 14.60	101.78 ± 14.38	96.10 ± 13.76	93.62 ± 13.59	90.45 ± 14.23	<0.001
CKD (*n*, %)	239 (2.63%)	38 (1.67%)	50 (2.23%)	64 (2.79%)	87 (3.81%)	<0.001
Overweight/obesity (*n*, %)	4,043 (44.45%)	1,040 (45.71%)	1,050 (46.79%)	994 (43.39%)	959 (41.97%)	0.004
Dyslipidemia (*n*, %)	3,805 (41.84%)	1,804 (79.30%)	709 (31.60%)	476 (20.78%)	816 (35.71%)	<0.001
Prediabetes (*n*, %)	5,283 (58.09%)	1,406 (61.80%)	1,352 (60.25%)	1,339 (58.45%)	1,186 (51.90%)	<0.001
T2DM (*n*, %)	2,068 (22.74%)	398 (17.49%)	429 (19.12%)	490 (21.39%)	751 (32.87%)	<0.001
Hypertension (*n*, %)	2,842 (31.25%)	716 (31.47%)	686 (30.57%)	717 (31.30%)	723 (31.64%)	0.872
CHD (*n*, %)	307 (3.38%)	84 (3.69%)	79 (3.52%)	70 (3.06%)	74 (3.24%)	0.638
Stroke (*n*, %)	63 (0.69%)	20 (0.88%)	12 (0.53%)	17 (0.74%)	14 (0.61%)	0.521
PAD (*n*, %)	10 (0.11%)	3 (0.13%)	1 (0.04%)	3 (0.13%)	3 (0.13%)	0.763
ASCVD (*n*, %)	315 (3.46%)	86 (3.78%)	80 (3.57%)	73 (3.19%)	76 (3.33%)	0.704

Data are mean ± SD. SD, standard deviation; Q, quartile; CHG index, cholesterol, high-density lipoprotein, and glucose index; BMI, body mass index; SBP, systolic blood pressure; DBP, diastolic blood pressure; TC, total cholesterol; TG, triglyceride; HDL-C, high-density lipoprotein cholesterol; LDL-C, low-density lipoprotein cholesterol; FBG, fasting blood glucose; PBG, 2 h postload blood glucose; HbA1c, glycated hemoglobin A1c; Cr, creatinine; eGFR, estimated glomerular filtration rate; CKD, chronic kidney disease; T2DM, type 2 diabetes mellitus; CHD, coronary heart disease; PAD, peripheral arterial disease; ASCVD, atherosclerotic cardiovascular disease.

### 3.2 Univariate analysis of variables contributing to prevalent CKD in the study population

Univariate analysis revealed that CHG index, age, AST/ALT, hypertension, T2DM, and ASCVD were positively correlated with the prevalence of CKD, whereas drinking, education level, BMR, and ALT were negatively correlated with the prevalence of CKD (*P* < 0.01 or *P* < 0.05; Table 2).

**TABLE 2:** Univariate analysis of variables contributing to chronic kidney disease (CKD) in study population.

Variables	B	OR (95% CI)	*P*
CHG index	0.796	2.217 (1.691–2.907)	0.000
Sex (female vs. male)	−0.209	0.811 (0.622–1.058)	0.123
Age	0.112	1.119 (1.103–1.134)	0.000
Smoking	0.029	1.029 (0.752–1.409)	0.856
Drinking	−0.359	0.698 (0.522–0.934)	0.016
Education level (≥9 years vs. < 9years)	−0.434	0.648 (0.473–0.888)	0.007
BMR	−0.002	0.998 (0.997–0.999)	0.000
HR	0.011	1.011 (1.000–1.022)	0.059
HbA1c/HDL-C	−0.032	0.968 (0.901–1.040)	0.380
GGT/HDL-C	−0.001	0.999 (0.995–1.004)	0.800
GGT	0.001	1.001 (0.998–1.003)	0.669
ALT	−0.020	0.981 (0.965–0.997)	0.017
AST	0.007	1.007 (1.000–1.014)	0.053
AST/ALT	0.251	1.286 (1.170–1.413)	0.000
Overweight/obesity	0.116	1.123 (0.865–1.458)	0.385
Hypertension	1.085	2.959 (2.279–3.842)	0.000
Dyslipidemia	0.123	1.131 (0.873–1.464)	0.352
T2DM	0.630	1.877 (1.433–2.459)	0.000
ASCVD	1.239	3.451 (2.244–5.307)	0.000

Data are expressed as OR (95% CI), unless stated otherwise. OR, odds ratio; CI, confidence interval.

### 3.3 Multivariable-adjusted ORs for the association of CHG index quartiles with risk of prevalent CKD

Table 3 shows the association between CHG index quartiles and the risk of prevalent CKD in Model 1–5 in study population. When analyzed as a continuous variable, each SD increase in the CHG index was associated with a 51% higher risk of prevalent CKD in Model 1 (OR = 1.510; 95% CI 1.312–1.737; *P* < 0.01). This association remained significant after full adjustment for sex, age, smoking, drinking, education level, BMR, HR, ALT, AST, GGT, GGT/HDL-C, AST/ALT, overweight/obesity, hypertension, dyslipidemia, HbA1c/HDL-C, T2DM, and ASCVD in Model 5 (OR = 1.577; 95% CI 1.301–1.911; *P* < 0.01). When the CHG index was analyzed by quartiles, the risk of prevalent CKD increased progressively across quartiles (all *P* for trend < 0.01). Compared with participants in the lowest quartile (Q1), those in the highest quartile (Q4) had significantly higher risk of prevalent CKD in Models 1–4 (*P* < 0.01 or *P* < 0.05). After additional adjustment for ASCVD, this association remained significant in Model 5 (OR: 1.328; 95% CI 1.069–1.648; *P* < 0.05), indicating the stability of the relationship between higher CHG index quartiles and increased risk of prevalent CKD.

**TABLE 3:** Association between cholesterol, high-density lipoprotein, and glucose (CHG) index quartiles and risk of prevalent chronic kidney disease (CKD).

Variable	Per SD increase	CHG index quartiles				*P* for trend
		Q1	Q2	Q3	Q4	
Model 1						
OR (95% CI)	1.510 (1.312–1.737)	1	1.342 (0.876–2.054)	1.301 (1.062–1.593)	1.326 (1.166–1.508)	<0.001
*P*-value	<0.001		0.176	0.011	<0.001	–
Model 2						
OR (95% CI)	1.515 (1.313–1.749)	1	1.009 (0.653–1.560)	1.357 (1.100–1.675)	1.331 (1.162–1.523)	<0.001
*P*-value	<0.001		0.108	0.004	<0.001	–
Model 3						
OR (95% CI)	1.513 (1.302–1.757)	1	1.372 (0.878–2.143)	1.356 (1.091–1.684)	1.324 (1.151–1.524)	<0.001
*P*-value	<0.001		0.165	0.006	<0.001	–
Model 4						
OR (95% CI)	1.561 (1.288–1.891)	1	1.196 (0.679–2.106)	1.286 (0.933–1.772)	1.309 (1.055–1.624)	0.004
*P*-value	<0.001		0.535	0.125	0.014	–
Model 5						
OR (95% CI)	1.577 (1.301–1.911)	1	1.208 (0.686–2.129)	1.290 (0.936–1.777)	1.328 (1.069–1.648)	0.003
*P*-value	<0.001		0.512	0.119	0.010	–

Data are expressed as OR (95% CI) + *P*-value, unless stated otherwise. Model 1: unadjusted; Model 2: adjusted for gender and age; Model 3: additionally adjusted for smoking, drinking, education level, BMR, and HR based on Model 2; Model 4: additionally adjusted for ALT, AST, GGT, GGT/HDL-C, AST/ALT, overweight/obesity, hypertension, dyslipidemia, HbA1c/HDL-C, and T2DM based on Model 3. Model 5: additionally adjusted for ASCVD based on Model 4.

### 3.4 Association of CHG index quartiles with increased risk of prevalent CKD in stratified analysis

Stratified analyses were performed to verify the stability of the relationship between CHG index quartiles and CKD. The results showed that the associations of higher CHG index quartiles with an increased risk of CKD prevalence were not consistently the same (Table 4). A significant association was observed only in participants who were men, non-smoker, non-drinker, aged ≥60 years, had less than a high school education (<9 years), overweight/obesity, T2DM, dyslipidemia, normal blood pressure, and no ASCVD (*P* for trend < 0.01 or *P* for trend < 0.05). Additionally, a significant interaction was detected between smoking status and CHG index quartiles in relation to CKD (*P* for interaction < 0.05).

**TABLE 4:** Association between cholesterol, high-density lipoprotein, and glucose (CHG) index quartiles and increased risk of chronic kidney disease (CKD) in different participants.

Variable	CHG index quartiles				*P* for trend	*P* for interaction
	Q1	Q2	Q3	Q4		
	OR (95% CI) *P*-value	OR (95% CI) *P*-value	OR (95% CI) *P*-value	OR (95%CI) *P*-value		
Gender						0.135
Men	1	1.321 (0.574–3.038) 0.513	1.467 (0.886–2.429) 0.137	1.810 (1.011–3.242) 0.046	0.037	–
Women	1	0.987 (0.442–2.201) 0.974	0.919 (0.579–1.460) 0.721	1.295 (0.962–1.743) 0.089	0.191	–
Age, years						0.174
<60	1	0.523 (0.156–1.756) 0.294	0.383 (0.170–0.861) 0.020	1.140 (0.696–1.865) 0.603	0.410	–
≥60	1	1.555 (0.819–2.953) 0.177	1.558 (1.111–2.185) 0.010	1.351 (1.060–1.722) 0.015	0.002	–
Smoking						0.025
No	1	1.300 (0.643–2.629) 0.465	1.423 (0.971–2.084) 0.070	1.574 (1.216–2.038) 0.001	0.001	–
Yes	1	1.003 (0.216–4.671) 0.997	0.592 (0.178–1.964) 0.391	1.067 (0.539–2.112) 0.852	0.772	–
Drinking						0.509
No	1	0.948 (0.519–1.733) 0.862	1.225 (0.881–1.702) 0.228	1.272 (1.014–1.595) 0.037	0.010	–
Yes	1	2.023 (0.651–6.291) 0.223	1.211 (0.580–2.527) 0.610	1.138 (0.701–1.847) 0.601	0.458	–
Education level						0.066
<9 years	1	1.854 (0.936–3.672) 0.077	1.632 (1.105–2.411) 0.014	1.458 (1.133–1.877) 0.003	0.003	–
≥9 years	1	0.302 (0.078–1.163) 0.082	0.757 (0.416–1.376) 0.361	0.973 (0.605–1.564) 0.909	0.413	–
Overweight/obesity						0.890
No	1	1.054 (0.467–2.376) 0.900	1.118 (0.716–1.745) 0.625	1.203 (0.894–1.618) 0.222	0.222	–
Yes	1	1.455 (0.638–3.317) 0.373	1.479 (0.912–2.399) 0.112	1.461 (1.063–2.009) 0.019	0.003	–
Gender						0.135
Glucose metabolism						0.062
NGT	1	0.728 (0.164–3.235) 0.677	1.186 (0.548–2.567) 0.666	1.017 (0.569–1.819) 0.954	0.368	–
Prediabetes	1	1.422 (0.629–3.217) 0.398	1.128 (0.665–1.914) 0.655	1.061 (0.699–1.611) 0.780	0.388	–
T2DM	1	1.971 (0.614–6.323) 0.254	1.719 (0.949–3.114) 0.074	1.673 (1.164–2.406) 0.005	0.005	–
Hypertension						0.057
No	1	0.730 (0.278–1.915) 0.522	1.571 (0.918–2.689) 0.100	1.455 (1.042–2.031) 0.028	0.006	–
Yes	1	1.591 (0.787–3.213) 0.196	1.036 (0.678–1.582) 0.871	1.279 (0.951–1.720) 0.104	0.195	–
Dyslipidemia						0.427
No	1	1.198 (0.487–2.949) 0.694	1.198 (0.745–1.925) 0.457	1.070 (0.771–1.484) 0.686	0.091	–
Yes	1	0.977 (0.458–2.083) 0.951	1.340 (0.870–2.062) 0.184	1.445 (1.094–1.909) 0.009	0.030	–
ASCVD						0.374
No	1	1.268 (0.697–2.308) 0.437	1.303 (0.927–1.832) 0.128	1.343 (1.070–1.686) 0.011	0.009	–
Yes	1	1.030 (0.499–2.125) 0.937	1.091 (0.286–4.167) 0.898	1.440 (0.540–3.841) 0.466	0.169	–

Data are expressed as OR (95% CI) + *P* value, unless stated otherwise. Adjusted for gender, age, smoking, drinking, education level, BMR, HR, ALT, AST, GGT, GGT/HDL-C, AST/ALT, overweight/obesity, hypertension, dyslipidemia, HbA1c/HDL-C, T2DM, and ASCVD.

### 3.5 The predictive value of CHG index in identify CKD in Chinese community-dwelling adults

**FIGURE 2:**
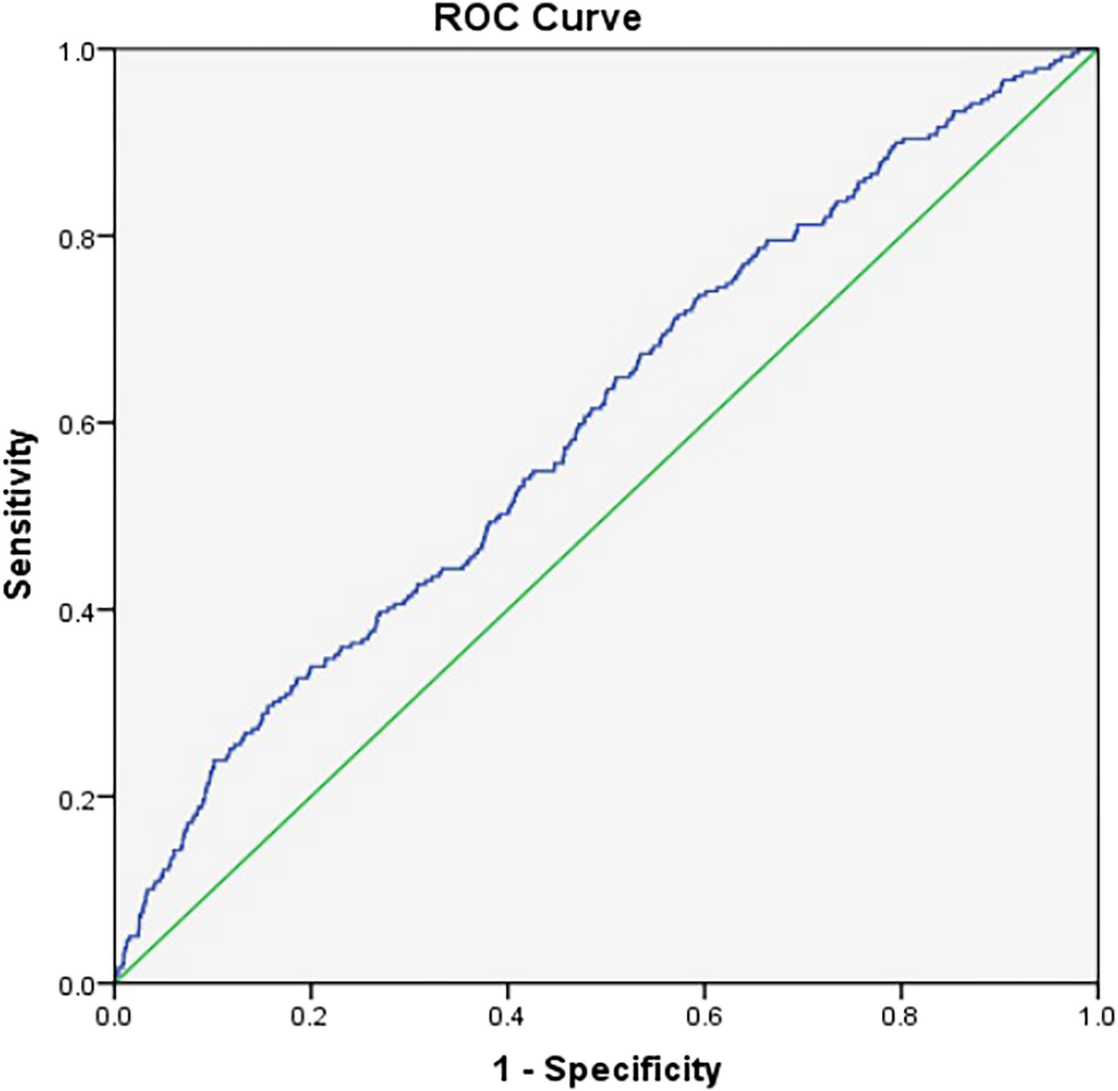
Receiver operating characteristics (ROC) curve analysis of cholesterol, high-density lipoprotein, and glucose (CHG) index to indicate CKD in Chinese community adults. The optimal cutoff point of the CHG index was 12.83 with a sensitivity of 73.6% a specificity of 40.6%, and the highest AUC equal to 0.601 (0.564–0.637, *P* = 0.000).

Receiver operating characteristics curve analysis was performed to evaluate the diagnostic performance of the CHG index for identification of CKD in Chinese community-dwelling adults. The results revealed that the optimal cutoff point for the CHG index in order to distinguish patients with CKD from those without was 12.83, with a sensitivity of 73.6%, a specificity of 40.6%, and a highest AUC equal to 0.601 (0.564–0.637, *P* < 0.01) (Figure 2).

## 4 Discussion

In this cross-sectional study, we for the first time observed that each SD increase in the CHG index and higher CHG index quartiles were significantly associated with an increased risk of prevalent CKD after extensive adjustments for various confounding factors. More important, the threshold of the CHG index for the presence of CKD was found to be 12.83. In addition, stratified analysis revealed that the association between a higher CHG index and increased risk of prevalent CKD was particularly evident in subgroups of men, non-smoker, non-drinker, individuals aged ≥60 years, those with less than a high school education, overweight/obesity, T2DM, dyslipidemia, normal blood pressure, and those without ASCVD.

Chronic kidney disease imposes substantial health burdens, with a high prevalence among middle-aged and elderly populations in Chinese communities, where effective disease-modifying interventions remain an unmet clinical need. Identifying early warning parameters for CKD is therefore of great importance. The CHG index, a novel metabolic composite indicator, was first proposed by Mansoori et al., who demonstrated through comparative analysis with the existing TyG index that the CHG index has a higher efficiency value for identifying T2DM (4). Subsequently, a cohort study including 6,249 adults aged 45 years and older demonstrated that an elevated CHG index is significantly correlated with an increased risk of CVD, and the CHG index is more effective than the TyG index in assessing CVD risk (3). CKD, T2DM, and CVD share common pathogenic pathways, such as IR, metabolic disturbance, and chronic inflammation (26); both the TyG and CHG indices are composite biomarkers that integrate blood lipid and glucose levels and may be associated with IR and chronic inflammation (3). Therefore, the CHG index may serve as a biomarker associated with CKD risk. In this cross-sectional study, we observed that the risk of prevalent CKD was increased progressively across CHG index quartiles, and participants in the highest quartile had a 67.3% higher odds of CKD compared with those in the lowest quartile after adjustment for all covariates. These findings are consistent with those of two previous studies (9, 10). A cross-sectional study of 175 Turkish patients with T2DM and a disease duration of ≥10 years showed that patients with diabetic nephropathy had elevated CHG index levels, and the CHG index was the only independent factor associated with diabetic nephropathy (9). Another retrospective study of 1,791 Chinese patients diagnosed with IgAN by renal biopsy reported that patients in higher quartiles of CHG index tended to have decreased eGFR, elevated serum Cr, and higher 24-h urine protein levels (10). Our findings further support that the CHG index is associated with the presence of CKD in Chinese community-dwelling adults, and may represent a promising candidate indicator for CKD in this population. More importantly, in the general middle-aged and elderly population of Chinese communities, we found that high CHG index indicated increased risk of CKD, and the CHG index was significantly associated with an increased risk of CKD, particularly in subgroups of men, non-smoker, non-drinker, individuals aged ≥60 years, those with less than a high school education, overweight/obesity, dyslipidemia, normal blood pressure, and those without ASCVD. These findings address a critical evidence gap regarding the relationship between the CHG index and CKD in underrepresented populations, and suggested that the CHG index may serve as a potential indicator for CKD in the general middle-aged and elderly Chinese community population.

The present study suggested that higher CHG index quartiles were significantly associated with an increased risk of prevalent CKD in men but not in women. Sex differences in the association between obesity and CKD have been described previously (27–29). Data from a Japanese study of 8,168 individuals who underwent general health screening reported that BMI had a graded association with low eGFR in men but not in women (27). In a 17-year cohort study, Iseki and colleagues also reported that elevations in BMI had a graded association with the cumulative incidence of ESRD compared with individuals with a baseline BMI of <21 kg/m^2^; however, this association was found only in men (28). Another study from Central South University, which included 26,655 patients, found that obesity was associated with urinary albumin-to-creatinine ration (UACR) in the male population but not in women (29). Together, these findings suggested a sex -specific association between the CHG index and CKD. The observed sex differences may be related to variations in sex hormones, adipokines, and lifestyle factors between men and women. It has been reported that estrogen possesses antioxidant properties that may reduce mesangial cell proliferation and delay glomerular fibrosis, whereas androgens may influence renal hemodynamics through activation of the renin–angiotensin–aldosterone system or by increasing proximal tubular reabsorption, and may stimulate the Fas/FasL-dependent apoptotic pathway, potentially contributing to renal tubular cell loss (29). Moreover, sexually dimorphic expression of adipocytokines has been described, with elevated adiponectin levels in females (30) and heightened pro-inflammatory mediators (resistin, TNF-α, IL-6) in males (31). This divergence functionally translates to adiponectin-mediated renoprotection against fibrotic injury, contrasted with the pro-fibrotic and pro-inflammatory renal damage potentiated by resistin and cytokines (32). Additionally, unhealthy lifestyles (e.g., cigarette smoking, excessive alcohol consumption) that are associated with CKD risk may be more prevalent in males (33). However, the mechanisms underlying these sex differences remain incompletely understood, and further research is warranted.

Lower educational attainment is a recognized risk factor for CKD (34, 35). In the present cross-sectional study, we also observed that lower educational attainment (<9 years) was positively correlated with prevalent CKD in the general middle-aged and elderly population of Chinese communities. Our findings are generally consistent with those of previous studies (34–36). Data from the Jackson Heart study of 3,430 African American men and women reported that individuals with higher education (defined as having at least an undergraduate degree) had substantially lower proportions of CKD and CKD risk factors compared with those at lower education levels (34). Findings from the Coronary Artery Risk Development in Young Adults (CARDIA) Study, which included 3,139 healthy young adults in the United States, showed that individuals with low educational attainment had a higher incidence of CKD over 20 years compared with those with medium and high educational attainment, and low educational attainment was significantly associated with a change in eGFR (35). Recently, a cross-sectional study of 3,212 adults aged 20–79 years reported that Chinese participants with CKD had a lower percentage of high school degrees, and low education level was associated with higher odds of albuminuria and CKD (36). Collectively, these findings further support that lower educational attainment is associated with higher CKD risk. The association between lower educational attainment and increased CKD risk may involve socioeconomic factors, unhealthy modifiable behaviors, and comorbid conditions (35, 36). Participants with lower educational attainment were less likely to have high income or health insurance, and were often unable to afford copays while living in communities with limited healthcare access (35). Previous studies have found that participants with low educational attainment lacked access to health information regarding disease awareness and prevention, and were more likely to have unhealthy modifiable behaviors including smoking, poor diet and nutrition, inadequate physical activity (35–37), and consequently have a higher prevalence of comorbid conditions including overweight/obesity, dyslipidemia, and T2DM—established factors associated with the presence and progression of CKD to ESRD (38–40). These observations are consistent with our finding that T2DM was correlated with prevalent CKD, and that a significant correlation between higher CHG index quartiles and higher risk of prevalent CKD was observed only in participants with overweight/obesity, T2DM, and dyslipidemia. This suggests that overweight/obesity, T2DM, and dyslipidemia—potentially associated with lower educational attainment—may act as mediating factors in the association between higher CHG index quartiles and higher risk of prevalent CKD. However, large-scale prospective cohort studies are needed to further explore these relationships.

Cigarette smoking, excessive alcohol consumption, hypertension, and ASCVD have been identified as risk factors for CKD (38–40). In the present cross-sectional study, we provided further evidence supporting the potential role of smoking, drinking, hypertension, and ASCVD in relation to CKD in the general middle-aged and elderly population of Chinese communities, as we observed significant associations between drinking, hypertension, ASCVD and prevalent CKD, as well as an interaction between cigarette smoking and the CHG index in relation to CKD. Unexpectedly, stratified analyses showed that significant associations between higher CHG index quartiles and CKD were detected only in non-smokers, non-drinkers, individuals with normal blood pressure, and those without ASCVD, but not in smokers, drinkers, individuals with hypertension and ASCVD. However, notably, counterintuitive results derived from subgroup analyses have been similarly reported in previous studies (41–45). In a population-based study of 9,436 participants aged ≥40 years, the fatty liver index (FLI) was significantly associated with elevated urinary albumin excretion and CKD in non-smokers and non-drinkers, but not in current smokers or drinkers (41).

Xue et al. reported that higher neck circumference quartiles were significantly associated with higher risk of decreased eGFR in female individuals without CVD but not in those with CVD (42). Moreover, findings from the China Cardiometabolic Disease and Cancer Cohort (4C) Study, which included 2,311 participants aged ≥40 years in Hubei Province, China, showed that the positive association between angiopoietin-like protein 8 levels and kidney function decline showed increased significance in individuals with no CVD. However, the association was diminished in individuals with CVD (43). Similarly, the REACTION study derived from the Luzhou city showed that positive associations between remnant cholesterol and lipid accumulation product quartiles and prevalent CKD were detected in individuals without CVD; however, these associations were attenuated in those with CVD (13, 44). Recently, a prospective cohort study of 5,719 Chinese community participants indicated a significant correlation between brachial-ankle pulse-wave velocity and major adverse cardiovascular events in the non-stable CAD and non-hypertension groups but not in the stable CAD and hypertension groups (45). These parallels our finding that the CHG index-CKD association is more pronounced in “healthier” populations, partly due to reduced competing risk from established cardiovascular risk factors. It is also possible that higher-risk populations may be aware of the hazards associated with smoking, alcohol consumption, hypertension, and ASCVD, therefore, may engage in more physical activity, modify their diet and lifestyle habits (e.g., quitting smoking, reducing alcohol intake, losing weight), and have better adherence to medical advice to effectively control blood lipids and blood pressure, as well as to prevent or delay the onset and progression of ASCVD and CKD. Additionally, several patients may be prescribed newer glucose-lowering medications such as sodium-dependent glucose transporters 2 (SGLT2) inhibitors or glucagon-like peptide-1 receptor agonists (GLP-1RA) receptor agonists due to obesity, diabetes, and heart failure, which have been shown to reduce ASCVD events and slow the progression of CKD. This could potentially contribute to the lack of a significant association between CHG index quartiles and CKD in these subgroups. However, further research in larger populations is needed to explore these observations.

## 5 Study strength and limitations

This study recruited a large general population from diverse communities in Luzhou City and, to our knowledge, was the first to investigate the association between the CHG index and prevalent CKD in the general population through comprehensive adjustment for covariates and stratified analyses. These represent key strengths of this study. However, several limitations should be acknowledged. First, the cross-sectional design of this study precludes establishing temporal or causal relationships between the CHG index and CKD. Thus, further prospective studies that allow us to evaluate the predictive power of the CHG index regarding CKD risk are needed in Chinese adults with different characteristics. Second, as only Chinese subjects were included in this study, our findings may not be generalizable to other ethnic groups, particularly those in developed or developing countries. Studies involving additional ethnic groups are needed to confirm these associations. Third, although multiple potential confounding factors, including smoking status, alcohol consumption, and education levels, were controlled in our analysis, the possibility remains that residual confounding variables and unmeasured factors (e.g., dietary patterns, lifestyle habits, and medications) may partially affect the observed association between the CHG index and CKD. To further strengthen the evidence, future studies should incorporate individual-level quantification of dietary and lifestyle factors, along with detailed medical records, when analyzing the relationship between the CHG index and CKD across different subgroups. Fourth, the subgroup analysis was exploratory, and the underlying mechanisms for these differential associations warrant further investigation in future studies. Fifth, our study lacked information on the UACR. UACR is an early and sensitive indicator of kidney damage, and albuminuria typically occurs earlier than elevations in serum Cr or declines in eGFR. Consequently, participants with normal eGFR who were considered free of CKD may have albuminuria and thus may have been misclassified, which may affect the results and limit the generalizability of the findings to CKD populations with UACR data available. Further prospective studies are needed to evaluate the association between the CHG index and CKD defined as low eGFR (eGFR < 60 ml/min/1.73 m^2^) and/or albuminuria (UACR ≥ 30 mg/g).

## 6 Conclusion

The present study demonstrated that a higher CHG index was significantly associated with higher risk of prevalent CKD among the general middle-aged and elderly population in Chinese communities, particularly in men, non-smokers, non-drinkers, individuals aged ≥60 years, those with less than a high school education, overweight/obesity, T2DM, dyslipidemia, and those without hypertension and ASCVD. In clinical practice, clinicians should be attentive to these relevant factors in a timely manner to help address the burden of CKD, based on the novel insights regarding the CHG index, which may serve as a convenient indicator for assessing individuals at high risk of CKD. Large, long-term interventional studies are needed to further investigate whether maintaining normal CHG index levels is associated with lower risk of prevalent CKD among Chinese general population.

## Data Availability

The original contributions presented in this study are included in the article/Supplementary materials, further inquiries can be directed to the corresponding authors.
