# Supplementary material for: Association of cholesterol, high-density lipoprotein, and glucose (CHG) index with chronic kidney disease in Chinese community adults: findings from the REACTION study
Source: Front Nutr. 2026 May 11;13:1778139. doi: 10.3389/fnut.2026.1778139 (PMC13199307; doi:10.3389/fnut.2026.1778139)
Supplement: Supplementary file 1 [file Table_1.doc]

**Supplementary Table 1 Metabolic and liver-related parameters across quartiles of the CHG index**

|  | **Total** | **Q1** | **Q2** | **Q3** | **Q4** |  |
| --- | --- | --- | --- | --- | --- | --- |
|  | **(n=9095)** | **(n=2275)** | **(n=2244)** | **(n=2291)** | **(n=2285)** | ***P*** |
|  |  | **≤12.60** | **12.61-12.94** | **12.95- 13.24** | **≥13.25** | **value** |
| BMR (kcal) | 1267.27±170.85 | 1297.17±183.86 | 1283.94±177.40 | 1261.63±165.87 | 1226.92±145.87 | <0.001 |
| HR (beats/minute ) | 79.91±11.11 | 78.82±10.66 | 79.23±10.89 | 80.18±10.86 | 81.41±11.84 | <0.001 |
| HbA1c/HDL-C | 5.21±1.90 | 6.94±2.01 | 5.27±1.33 | 4.54±1.18 | 4.12±1.61 | <0.001 |
| GGT/HDL-C | 23.10±32.89 | 23.11±30.10 | 24.14±32.75 | 21.94±25.07 | 23.22±41.46 | <0.001 |
| GGT (U/L) | 27.45±42.33 | 19.86±26.57 | 26.51±32.21 | 28.28±33.74 | 35.04±64.38 | <0.001 |
| ALT (U/L) | 15.46±11.44 | 12.98±9.92 | 15.49±10.86 | 16.24±12.86 | 16.86±11.39 | <0.001 |
| AST (U/L) | 21.36±11.64 | 16.90±9.20 | 21.03±9.24 | 22.97±12.90 | 24.50±13.11 | <0.001 |
| AST/ALT | 1.66±0.78 | 1.58±0.71 | 1.63±0.66 | 1.70±0.87 | 1.72±0.84 | <0.001 |

Data are mean ± SD. SD, standard deviation; Q, quartile; CHG index, cholesterol, high-density lipoprotein, and glucose index; BMR, basal metabolic rate; HR, heart rate; HbA1c/HDL-C, glycated hemoglobin to high-density lipoprotein cholesterol ration; GGT/HDL-C, glutamyl transpeptidase to high-density lipoprotein cholesterol ratio; GGT, glutamyl transpeptidase; ALT, alanine aminotransferase; AST, aspartate aminotransferase; AST/ALT, aspartate aminotransferase to alanine aminotransferase ratio.
